# Engineering *Saccharomyces cerevisiae* for the production of natural osmolyte glucosyl glycerol from sucrose and glycerol through Ccw12-based surface display of sucrose phosphorylase

**DOI:** 10.1186/s13036-024-00468-7

**Published:** 2024-11-22

**Authors:** Tea Martinić Cezar, Nenad Marđetko, Antonija Trontel, Antonia Paić, Anita Slavica, Renata Teparić, Bojan Žunar

**Affiliations:** 1 Laboratory for Biochemistry, Department of Chemistry and Biochemistry, University of Zagreb Faculty of Food Technology and Biotechnology, Pierottijeva 6, Zagreb, 10000 Croatia; 2 Laboratory for Biochemical Engineering, Industrial Microbiology and Malting and Brewing Technology, Department of Biochemical Engineering, University of Zagreb Faculty of Food Technology and Biotechnology, Pierottijeva 6, Zagreb, 10000 Croatia

**Keywords:** Glucosyl glycerol, Osmolyte, Sucrose phosphorylase, *Saccharomyces cerevisiae*, Surface display, Ccw12

## Abstract

**Background:**

Yeast *Saccharomyces cerevisiae* is widely recognised as a versatile chassis for constructing microbial cell factories. However, producing chemicals from toxic, highly concentrated, or cell-impermeable substrates, or chemicals dependent on enzymatic reactions incompatible with the yeast’s intracellular environment, remains challenging. One such chemical is 2-*O*-(α-D-glucopyranosyl)-*sn*-glycerol (glucosyl glycerol, αGG), a natural osmolyte used in the cosmetics and healthcare industries. This compound can be synthesised in a one-enzyme reaction from sucrose and glycerol by *Leuconostoc mesenteroides* sucrose phosphorylase (SucP), an enzyme which, in a low-water, glycerol-rich, phosphate-free environment, transfers the glucosyl moiety from sucrose to glycerol.

**Results:**

In this study, we engineered a yeast microbial cell factory for αGG production. For this purpose, we first focused on the abundant yeast GPI-anchored cell wall protein Ccw12 and used our insights to develop a miniature Ccw12-tag, which adds only 1.1 kDa to the enzyme of interest while enabling its covalent attachment to the cell wall. Next, we Ccw12-tagged SucP and expressed it in an invertase-negative strain of yeast *S. cerevisiae* from the *PHO5* promoter, i.e., promoter strongly induced under phosphate-free conditions. Such SucP isoform, covalently C-terminally anchored to the outer cell surface, produced extracellularly 37.3 g l^− 1^ (146 mM) of αGG in five days, while the underlying chassis metabolised reaction by-products, thereby simplifying downstream processing.

**Conclusions:**

The here-described *S. cerevisiae* strain, displaying C-terminally anchored sucrose phosphorylase on its cell surface, is the first eukaryotic microbial cell factory capable of a one-step αGG production from the readily available substrates sucrose and glycerol.

**Supplementary Information:**

The online version contains supplementary material available at 10.1186/s13036-024-00468-7.

## Background

Yeast *Saccharomyces cerevisiae* is widely recognized as a versatile and valuable chassis for constructing microbial cell factories [1]. However, using it to implement certain bioprocesses can be challenging, especially if the bioprocesses require toxic, highly concentrated, or cell-impermeable substrates or if the desired enzymatic reaction proceeds only under conditions incompatible with the yeast’s intracellular environment [2, 3].

One such bioprocess is the one-step production of 2-*O*-(α-D-glucopyranosyl)-*sn*-glycerol (glucosyl glycerol, αGG), a commercially relevant osmolyte comprised of a glucose and glycerol moiety [4]. αGG is a heteroside naturally produced by salt-tolerant cyanobacteria [5], known for its low hygroscopicity, high water-retaining capacity, excellent biocompatibility, and ability to protect macromolecules from damage caused by harsh environments. Because of these properties, αGG is utilized in a variety of sectors, including cosmetics, healthcare, food industry, and enzyme production. However, producing αGG via the cyanobacterial two-enzyme metabolic pathway, involving αGG-phosphate synthase and αGG-phosphate phosphatase, is costly, as the reaction depends on energy-rich ADP-glucose and glycerol-3-phosphate [6]. A more cost-effective approach employs a one-step bioprocess using the enzyme sucrose phosphorylase (SucP) [7].

SucP is a glucosyl transferase that primarily catalyses the transfer of the glucosyl moiety from sucrose to phosphate, thus producing glucose-1-phosphate and fructose. However, due to its substrate promiscuity, SucP can also use glycerol or water as acceptor molecules in the absence of phosphate, thus producing αGG or merely hydrolysing sucrose into glucose and fructose, respectively. As such, acting on cheap substrates sucrose and glycerol, SucP can catalyse one-step αGG synthesis in a low-water, phosphate-free environment [8], a reaction for which it requires no cofactors. Typically, this reaction is not performed with living cells as intracellular space abounds with water, is phosphate-rich, and has very limited pools of sucrose and glycerol available. Instead, the reaction employs chemically immobilized enzymes [9]. An alternative approach to performing this reaction would rely on a protein engineering technique known as surface display, therefore relocating this reaction to the outer cell surface [10, 11]. Such an approach would direct SucP through the secretion pathway to the cell wall, where it would be immobilized near the catalytically active enzyme’s C-terminus, thus transforming the cell wall into a self-sustaining, αGG-producing catalyst.

Surface display typically involves the covalent attachment of secreted enzymes to the cell surface, to help prevent their loss into the surrounding environment. Cells utilize two main mechanisms for such covalent binding to the cell wall, employing N-terminal Pir repeats or C-terminal glycosylphosphatidylinositol (GPI)-signal sequences [12]. Proteins equipped with Pir repeats can directly attach to the β-1,3-glucan in the cell wall via glutamate residues present in these repeats [13]. These proteins generally have Pir repeats near their N-termini and are epitomized by the Pir protein family [14]. Leveraging this mechanism, we recently developed a Pir-tag [15], a 4.5 kDa peptide designed for the N-terminal anchoring of target proteins to the cell surface, aiming to enhance their stability and functionality on the cell surface.

The cell utilizes another mechanism to covalently bind some proteins near the proteins’ C-terminus, one involving a GPI anchor. Such binding to β-1,6-glucan involves the sequential actions of GPI transamidase [16] and the Dcw1 and Dfg5 transglycosylases [17]. These enzymes specifically recognize a glycine residue located within the GPI signal sequence at the protein’s C-terminus, facilitate the transfer of a GPI anchor to this site, and covalently bind the protein to β-1,6-glucan. Such C-terminal anchoring is helpful when N-terminal anchoring destabilizes or completely inactivates the protein of interest, as preliminary results indicated for SucP [18]. While examples of C-terminal anchoring have been published [19, 20], a small tag that would incorporate all features essential for efficient C-terminal anchoring has yet to be developed.

In this work, we developed a C-terminal anchoring tag based on *S. cerevisiae* protein Ccw12 and utilized it to display the enzyme SucP from *Leuconostoc mesenteroides* on the yeast outer cell surface. Initially, we analysed the structure of the cell wall protein Ccw12 and, through a nitrocefin assay, identified the minimal Ccw12 region necessary for constructing an efficient Ccw12-tag. This tag was then employed to anchor SucP to the cell surface, resulting in a C-terminally anchored cell wall SucP isoform that produced αGG extracellularly. Finally, we determined the kinetic parameters of such SucP reaction, noting how they are modified by the metabolism of the underlying host cell.

## Methods

### Prediction of protein structures

Protein sequences were aligned with the Smith-Waterman algorithm [21], as implemented in SnapGene [22], using standard parameters. The 3D protein structures, along with their confidence scores, were computed using AlphaFold 3 [23], accessed via the AlphaFold Server. The structures were visualised in PyMOL [24], employing the cealign command to superpose 3β-strand motifs. Additional yeast proteins with similar motifs were identified using Dali [25] by searching against the AlphaFold 2 database of the entire *S. cerevisiae* proteome [26].

### Media and growth conditions

NEB Stable *E. coli* were cultured overnight at 37 °C, with shaking at 200 rpm in liquid 2xYT media (16.0 g/l tryptone, 10.0 g/l yeast extract, 5.0 g/l NaCl) or on LB plates (10.0 g/l tryptone, 5.0 g/l yeast extract, 5.0 g/l NaCl, 15.0 g/l agar) with 100 µg/ml ampicillin. *S. cerevisiae* strains BY 4741 and SEY 6210 were grown at 30 °C with shaking at 180 rpm in YPD medium (20.0 g/l peptone, 10.0 g/l yeast extract, 20.0 g/l glucose, and 20.0 g/l agar for solid media). *S. cerevisiae* strains carrying reporter plasmids were cultured in chemically defined media without histidine (-his: 6.70 g/l Difco Yeast nitrogen base without amino acids, 20.0 g/l glucose, 1.6 g/l drop-out without histidine, and 20.0 g/l agar for solid media). To induce the *PHO5* promoter, cells were initially grown in a repressive medium with 1 g/l KH_2_PO_4_, followed by growth in a phosphate-free -his medium as in Martinić Cezar, et al. [15]. For αGG-producing reactions, 10 OD_600_ of induced SEY 6210 cells were mixed with MOPS buffer (250 mM, pH 7), sucrose (0.9 M), and glycerol (2 M), to a final volume of 0.5 mL and incubated at 30 °C/300 rpm/20 h, unless noted otherwise. After the incubation, the cells were pelleted, and the supernatant was filtered and stored at -20 °C until the UPLC analysis. The effect of pH was investigated in 250 mM Na-citrate (pH 5), Na-citrate (pH 6), MOPS (pH 7), and Tris-HCl (pH 8) buffers. All growth experiments were performed in triplicates.

### Plasmid and strain construction

Plasmid construction was performed in NEB Stable *E. coli* using restriction cloning, Q5 polymerase, and HiFi Assembly (New England Biolabs, Frankfurt am Main, Germany), as described previously [27]. *S. cerevisiae* strains were transformed using the method described by Gietz, et al. [28]. Additional construction details are available in the Supplementary material. Plasmid DNA was purified using the NucleoSpin Plasmid Mini Kit and extracted from agarose gels with the NucleoSpin Gel and PCR Clean-up Kit (Macherey-Nagel, Duren, Germany). All constructs were confirmed by restriction digest and Sanger sequencing, as described previously [29], with results matching the computed restriction maps. Primer synthesis was outsourced to Macrogen Europe (Amsterdam, Netherlands), and Sanger sequencing services were provided by Microsynth (Balgach, Switzerland).

### Isolation of cell wall proteins

Cell wall proteins were isolated following the methodology described by Lozančić, et al. [30]. Briefly, cells were cultured in a phosphate-free -his medium until they reached the stationary phase, then resuspended in KP8 buffer (potassium phosphate buffer, 50 mM, pH 8). The cells were mechanically disrupted using glass beads and washed four times with KP8 buffer. Non-covalently bound proteins were extracted by boiling the cell wall fraction twice in reducing Laemmli buffer (0.0625 M Tris, pH 6.8, 2% SDS, 5% v/v β-mercaptoethanol, 0.001% bromophenol blue), which released non-covalently bound proteins to the supernatant. To isolate covalently bound proteins, the cell walls stripped of the non-covalently bound proteins were washed three times with KP8 buffer and once with KP6 buffer (potassium phosphate buffer, 50 mM, pH 6). The remaining solid fraction was weighed and resuspended in KP6 buffer at the ratio of 1 mg of stripped cell walls to 1 µl of KP6 buffer. To this suspension, 3 µg of enzyme Laminarinase 81 A from *Clostridium thermocellum* (NZYtech, Lisbon, Portugal) was added to each 25 µl of cell wall suspension, and the mixture was incubated at 55 °C/2 h. Following centrifugation, the protein-containing supernatant was collected. The protein samples of non-covalently and covalently bound proteins were standardized by adjusting the volume of the extract used for electrophoresis relative to the mass of the wet and stripped cell walls measured before and after the extraction process, respectively.

### Immunoblotting

Isolated proteins were resolved on 10% SDS-PAGE gels, then transferred to nitrocellulose membranes using the semidry Trans-Blot Turbo™ Transfer System (Bio-Rad, Hercules, United States). The proteins were analysed by immunoblotting, following standard procedures detailed in Novačić, et al. [31]. Blots were developed using Clarity Western ECL substrates (Bio-Rad, Hercules, United States) and visualized with a C-DiGit Blot scanner (LI-COR Biosciences, Lincoln, United States). The isolated cell wall proteins were probed with an anti-HA peroxidase-conjugated antibody at a dilution of 1:1,200 (11667475001, Roche, Basel, Switzerland).

### Nitrocefin assay

The nitrocefin assay was performed as described previously [32]. Cells were inoculated into 5 ml of -his medium supplemented with KH_2_PO_4_ and cultured overnight at 30 °C with shaking at 180 rpm. The next day, cells in the stationary phase were diluted to 0.5 OD_600_/ml in 15 ml of the same medium and grown until they reached 2 OD_600_/ml. To induce the *PHO5* promoter, the cells were washed with sterile deionized water (sdH_2_O), diluted to 0.3 OD_600_/ml in 15 ml of phosphate-free -his medium, and incubated overnight at 30 °C/180 rpm, reaching approximately 2 OD_600_/ml by morning. Such cells were washed in sdH_2_O, then in KP7 buffer (potassium phosphate buffer, 50 mM, pH 7), and resuspended in the same buffer at a concentration of 100 OD_600_/ml. For the activity assay, 0.05 OD_600_ of cells was resuspended in 475 µl of KP7 buffer, shaken at 30 °C/1,200 rpm/2 min, mixed with 25 µl of nitrocefin (1 mM, dissolved in KP7 buffer with 5% DMSO), and incubated at 30 °C/1,200 rpm/5 min. The reaction was stopped by centrifugation (8,000 rpm/30 s), and the absorbance of the supernatant was measured at 482 nm. Each strain’s activity was assessed in technical triplicates and at least biological duplicates.

### Ultra-high performance liquid chromatography

To determine the concentrations of αGG, glucose, fructose, sucrose, and glycerol, 1.0 ml of appropriately diluted supernatant from the reaction mixture was filtered through a 0.20-µm nylon syringe filter (LLG, Meckenheim, Germany) into a UPLC glass vial. The analysis was conducted on Agilent Technologies 1290 Infinity II LC system (Santa Clara, CA, United States) equipped with a SUGAR precolumn (4 mm × 3 mm; Phenomenex, Des Plaines, IL, United States) and a Cosmosil Sugar-D column (15 cm × 4.6 mm; Nacalai Tesque, Kyoto, Japan), with a refractive index detector. The mobile phase consisted of a water and acetonitrile mixture (75:25). The injected sample volume was 10 µl, and the flow rate was set at 0.6 ml/min, with the column temperature maintained at 30 °C. Standard retention times were determined with αGG (AdipoGen Life Sciences, San Diego, USA), glucose (Lach-Ner, Czech Republic), fructose (Sigma-Aldrich, Germany), sucrose (Sigma-Aldrich, Germany), and glycerol (Thermo Fisher Scientific, USA). Data analysis was conducted using OpenLab CDS software. The K_M_ values were determined based on the Lineweaver-Burke diagrams.

## Results

We implemented one-step αGG production in *S. cerevisiae* by expressing in yeast cells *L. mesenteroides* SucP, which can transfer the glucosyl moiety from sucrose to glycerol. However, this reaction occurs only in a low-water, high-glycerol, phosphate-free medium, incompatible with the intracellular environment of live cells. To overcome this, we employed surface display, binding SucP to the cell’s outer surface. To enhance the enzyme’s stability while preserving its activity, we anchored it C-terminally to the cell wall. Since existing literature did not describe a suitable mini-tag for this purpose, we first developed one based on the abundant C-terminally anchored cell wall protein Ccw12.

### The structure of Ccw12

To develop a tag that would direct proteins to the cell wall and covalently anchor them to it at their C-termini, we focused on the cell wall protein Ccw12. This protein was selected due to its small size, strong expression and high abundance, which suggested the cell would be highly efficient at secreting its derivatives to the cell surface. Ccw12, spanning 133 amino acid residues, is extensively N- and O-glycosylated and binds covalently to the cell wall’s β-1,6-glucan through the C-terminally located GPI anchor [12]. This protein is highly expressed, at 14,000-170,000 copies per cell [33] and encoded by a gene with an exceptionally high codon adaptation index (0.87) [34]. While the exact function of Ccw12 is not yet fully understood, it is known that the protein localises to the areas of active cell wall synthesis, e.g., future bud sites and the septum, where it helps to stabilize the cell wall [35].

**Fig. 1:**
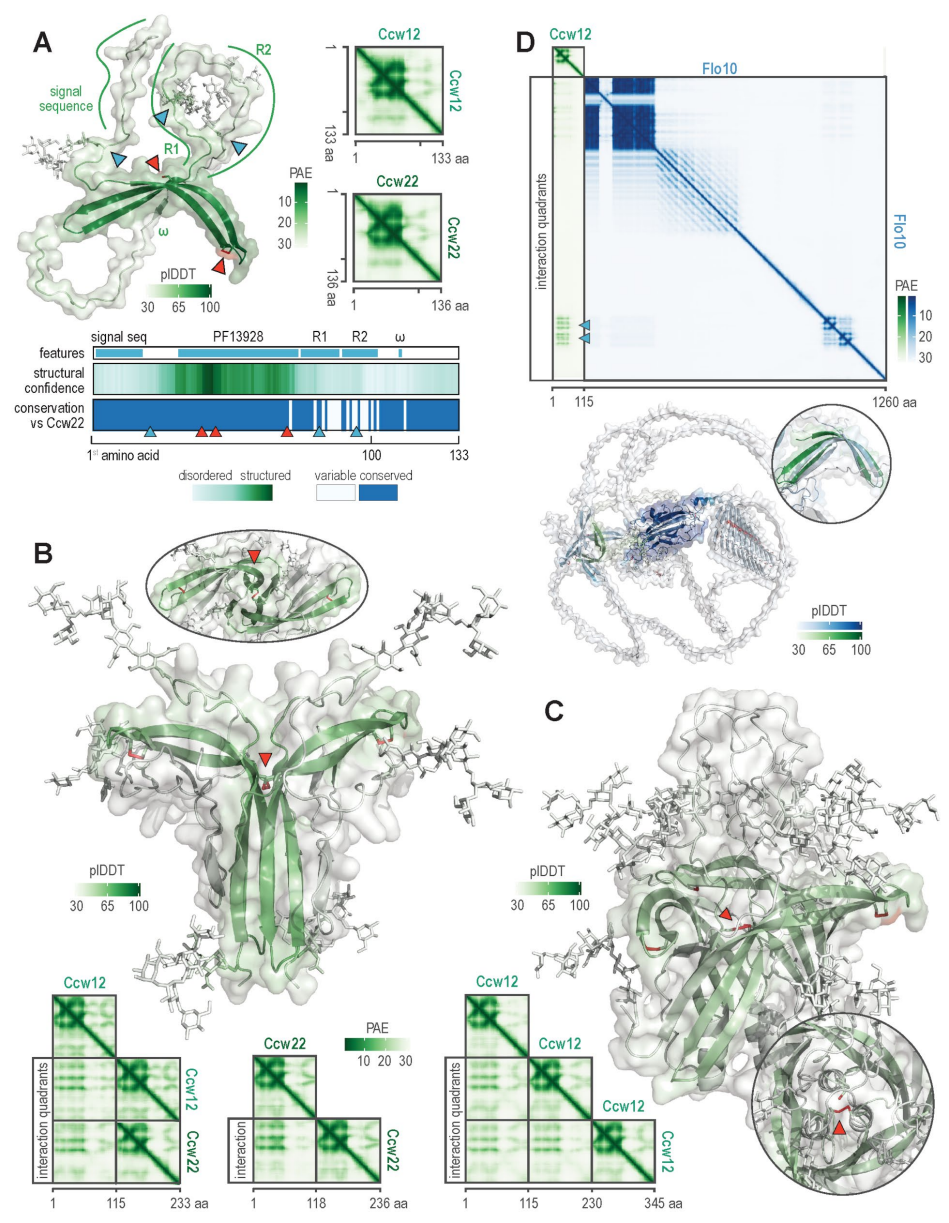
Alphafold 3 modelling of *S. cerevisiae* protein Ccw12. (**A**) The predicted 3D structure of Ccw12. The structure is colour-coded to reflect local confidence scores per amino acid residue (plDDT), with light green indicating low confidence and dark green indicating high confidence. Blue triangles denote N-glycosylated asparagines linked to core glycans. Red triangles mark three conserved cysteine residues, two of which form a disulphide bond. On the right, predicted aligned error (PAE) diagrams for Ccw12 and Ccw22 illustrate global interaction confidence between amino acid residues, with light green denoting low confidence and dark green denoting high confidence. Below the 3D model, the uppermost track outlines the Ccw12 primary sequence, showing the signal sequence, the Pfam domain PF13928 (which folds into a flocculin type 3 repeat), Ccw12-characteristic repeats R1 and R2, and the GPI transamidase-targeted ω-site. The second track correlates these features to their plDDT scores, while the last track indicates sequence similarity to Ccw22, with blue lines marking identical or similar residues. (**B**) Homodimer formation. Ccw12 can potentially form homodimers, in which two monomers link through the C72-C72 interchain disulphide bond, indicated by the red arrow and accentuated in the ellipsoid inset showing a top-down view of the homodimer. The bottom PAE diagrams show Alphafold 3 predicts the formation of Ccw12 and Ccw22 homo- and heterodimers with similar confidence. (**C**) Homotrimer formation. Ccw12 might also form homotrimers, in which three C72 residues group together, with two forming an interchain disulphide bond. Such cysteine grouping is highlighted by the red arrow and in the top-down view of the complex in the circular inset. **(D**) Forming heterodimers with other cell wall proteins. Ccw12 potentially forms heterodimers with other cell wall proteins that carry the 3β-strand motif. The panel shows the PAE diagram between the cell wall proteins Ccw12 and Flo10, with grey triangles highlighting potential protein-protein interactions between the motifs. The circular inset displays aligned 3β-strand motifs from Ccw12 (green) and Flo10 (blue), emphasizing their similarity, while the bottom 3D structure depicts the predicted Ccw12-Flo10 complex

To identify the key structural features of Ccw12, we modelled it with Alphafold 3, a deep neural network designed to accurately predict biomolecular structures, including those of post-translationally modified proteins and their complexes [23]. Alphafold 3 modelled N-glycosylated Ccw12 as a glycoprotein whose structure is dominated by three antiparallel β-strands (3β-strand motif) encoded by the flocculin type 3 repeat (Pfam domain 13928). Non-structured regions extend before and after this motif, encompassing the signal sequence, Ccw12-characteristic repeats R1 and R2, and the C-terminal tail containing the ω-site (Fig. 1A). At the ω-site, GPI transamidase cleaves the polypeptide chain and covalently attaches the GPI anchor to the newly exposed C-terminal residue [36]. The 3β-strand motif is distinctly visible on the predicted aligned error (PAE) diagram, a graph denoting Alphafold 3’s interaction confidence [37], on which it produces a diamond-like pattern, characteristic for regions with high-confidence structures. This motif encompasses the three Ccw12’s conserved cysteine residues (C40, C45, and C72) [34], the first two of which form a disulphide bond that stabilizes the β-turn between them. The Ccw12’s three N-glycosylation sites (N21, N81, and N97) [38] are located in non-structured regions, immediately after the signal sequence and within Ccw12 repeats R1 and R2. Thus, Ccw12 is a small, partially unstructured, N-glycosylated, C-terminally anchored cell wall protein whose structure is primarily defined by the 3β-strand motif stabilized through a disulphide bond.

We compared Ccw12 to its paralog Ccw22 (Fig. 1A), which emerged during yeast whole genome duplication 100–150 million years ago [39, 40]. Structurally, Ccw22 closely resembles Ccw12; both proteins share the 3β-strand motif, the diamond-like PAE pattern, and 81% amino acid sequence identity. However, the two proteins diverge significantly in the regions encoding the R1 and R2 repeats, with Ccw22 even lacking the third N-glycosylation site. Thus, Ccw12 and Ccw22 have almost identical core structures but differ in their repeat regions.

The high conservation of the 3β-strand motif in both Ccw12 and Ccw22 suggested that this structural feature might be functional, potentially facilitating the formation of protein-protein complexes. We first investigated whether Ccw12 and Ccw22 could form homo- and heterodimers. Alphafold 3 indicated a significant potential for such structures, in which the 3β-strand motifs from the two polypeptide chains are linked via hydrogen bonds and a disulphide bond between their phylogenetically conserved C72 residues (Fig. 1B). In such dimers, the N-glycosylated regions extend away from the well-defined central structure. The interaction quadrants in the PAE diagrams suggested both proteins are equally likely to form homo- and heterodimers. Alphafold3 also successfully modelled the Ccw12 trimer (Fig. 1C), in which all three C72 residues group together, with two of them forming a disulphide bond. Thus, Ccw12 and Ccw22 likely form N-glycosylated dimers and trimers on the cell surface, stabilized by interchain hydrogen and disulphide bonds.

**Fig. 2:**
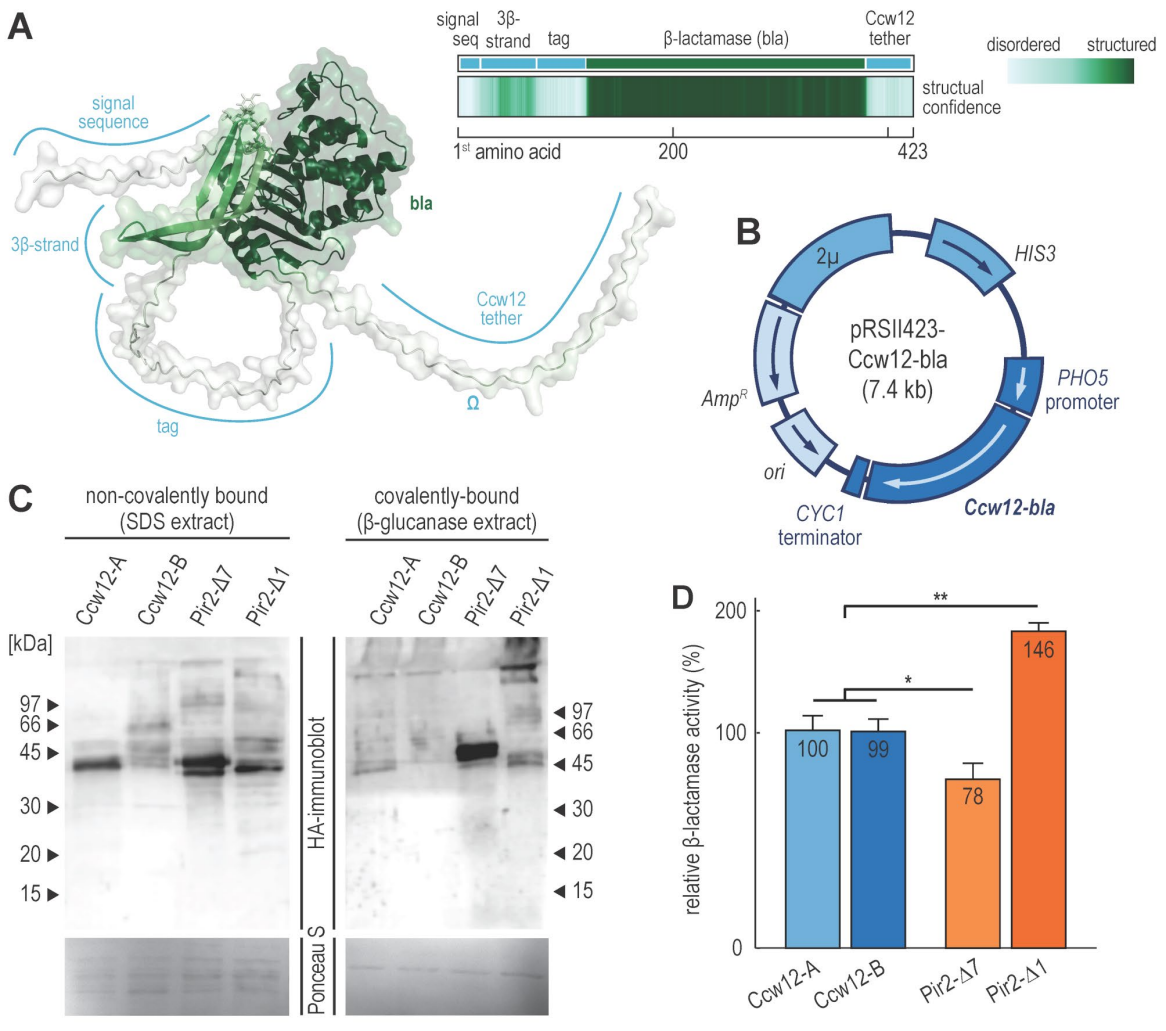
Ccw12-β-lactamase fusion proteins. (**A**) The predicted 3D structure of fusion reporter protein Ccw12-β-B. The 3D structure is colour-coded according to the plDDT score and labelled to highlight structural features. The tracks on the right correlate these features with their locations in the primary sequence and their corresponding plDDT scores. (**B**) Map of a yeast shuttle vector encoding Ccw12-β-lactamase fusion proteins. *Amp*^*R*^ – gene conferring ampicillin resistance to *E. coli*, *ori* – *E. coli* plasmid replication origin, *2µ* – yeast 2µ plasmid replication origin, *HIS3* – yeast selective marker allowing *his3Δ* strains growth on media lacking histidine, *PHO5* promoter – yeast promoter induced in phosphate-free medium, *Ccw12-bla* – open reading frame encoding Ccw12-β-lactamase fusion protein, *CYC1* terminator – terminator of yeast *CYC1* gene. (**C**) Anti-HA immunoblotting of Ccw12- and Pir2-fusion proteins. The immunoblot shows non-covalently and covalently bound Ccw12- and Pir2-fusion proteins. The lower membrane, stained after electrotransfer with Ponceau S, serves as a loading control. (**D**) Relative β-lactamase activities of Ccw12- and Pir2-fusion proteins. The graph displays the activities normalised to Ccw12-β-A, with error bars indicating standard deviations. *p-value < 0.05, **p-value < 10^− 6^

We next investigated whether a similar 3β-strand motif was present in any other *S. cerevisiae* proteins, as through it, Ccw12 and Ccw22 could interact with them. Using Dali, a web server for comparing protein structures [41], we found significant 3D similarity (Z-value > 2) between Ccw12’s 3β-strand motif and Alphafold 2-predicted structures of GPI-anchored cell wall proteins Flo1, Flo5, Aga1, Flo9, Flo10, protein kinase Gcn2, mitochondrial protein Som1, splicing factor Prp19, and uncharacterised proteins Yer145c-a, Ykr005c, and Yil012w. All five listed cell wall proteins carried the diamond-like PAE patterns characteristic of the 3β-strand motif. Among them, Flo5, Flo9, and Flo10 also encoded multiple flocculin type 3 repeats, as did Fig. 2, which is a paralog of Aga1. Additionally, sequence analysis revealed that Ykr005c encodes a signal sequence (likelihood: 0.9993, SignalP-6.0) [42], suggesting it localises to the cell wall. Thus, the 3β-strand motif of Ccw12 is also present in several other GPI-anchored cell wall proteins, primarily involved in cell-carbohydrate or cell-cell interactions.

To investigate the interaction hypothesis further, we focused on the potential Flo10-Ccw12 and Aga1-Ccw12 interactions. Structural alignment with PyMOL showed that Flo10 contained a 3β-strand motif very similar to that of Ccw12 (Fig. 1D). AlphaFold 3 modelling also indicated that the two Flo10 3β-strand motifs and one Ccw12 motif could interact to form a protein-protein complex. Moreover, Alphafold 3 modelling of the Aga1-Aga2-Ccw12 complex suggested Ccw12 through residue C72 forms an interstrand disulphide bond with Aga1 (Supplementary Fig. 1). Thus, the 3β-strand motif of Ccw12 and its C72 residue likely facilitate diverse protein-protein interaction.

### Constructing a minimal Ccw12-tag

After determining the potentially relevant cell-wall anchoring regions, we used portions of its sequence to develop a Ccw12-based minimal anchoring tag, aiming to C-terminally tether the protein of interest to the outer cell surface. For this purpose, we constructed Ccw12-β-lactamase fusion proteins, following a strategy similar to the one we previously employed to develop an efficient N-terminally anchored Pir-based surface display [15]. This approach involved using β-lactamase as a cell wall-targeted reporter enzyme that cleaves nitrocefin, a cell-membrane impenetrable penicillin analogue whose absorbance spectrum changes once its β-lactam ring is cleaved. Thus, the nitrocefin assay enabled us to quantify only the β-lactamase secreted to the cell wall.

We constructed two Ccw12-β-lactamase fusion proteins, Ccw12-β-A and Ccw12-β-B. Both proteins contained the Ccw12 signal sequence (1–20 aa of Ccw12), a triple HA-tag, a His-tag, a codon usage-optimised β-lactamase, and the Ccw12 C-terminus (104–133 aa of Ccw12), which contained the ω-site necessary for covalent tethering to the cell wall (Fig. 2A). Additionally, Ccw12-β-B featured the 50 amino acid-long, well-structured 3β-strand motif of Ccw12, inserted between the signal sequence and the triple HA-tag. The full-length, unprocessed chains of Ccw12-β-A and Ccw12-β-B had molecular weights of 40.2 kDa and 45.4 kDa, respectively. The genes encoding these fusion proteins were carried on a yeast 2µ *HIS3* shuttle vector and regulated by the *PHO5* promoter and *CYC1* terminator (Fig. 2B). This setup allowed for controlled protein induction in a phosphate-free medium and thereby avoided the potential growth defects in the exponential phase due to an oversaturated protein secretion system. Moreover, it induced the expression of cell wall-targeted proteins in the growth phase, during which the cell wall was already quite robust and compatible with the reaction conditions required by SucP.

We first tested whether the cells could secrete and bind the Ccw12-β-lactamase fusion proteins to their cell wall. We induced the *PHO5* promoter, isolated non-covalently and covalently bound cell wall proteins, i.e., SDS and β-glucanase protein fractions of the cell wall, respectively, and performed an anti-HA immunoblot. The immunoblot of the non-covalently bound fraction revealed that the cells secreted both fusion proteins with similar efficiency, and it displayed multiple Ccw12-β-B bands, likely due to heterogenous O-glycosylation (Fig. 2C). Thus, both Ccw12-β-A and Ccw12-β-B were successfully secreted to the cell wall, although Ccw12-β-A was probably more homogenously glycosylated.

We also compared the covalent and non-covalent cell wall binding of the Ccw12-β-lactamase fusion proteins to that of previously developed Pir-based reporters Pir2-Δ7 and Pir2-Δ1 (Fig. 2C). These Pir-based reporters featured a minimal Pir-tag and a larger portion of the Pir2 protein that enhanced the efficiency of the surface display, respectively. The comparison revealed that the most prevalent isoforms of all four reporter proteins were of approximately similar size. Furthermore, it showed that the secretion level of Ccw12-β-A was comparable to that of Pir2-Δ7 and Pir2-Δ1, whereas Ccw12-β-B was secreted less efficiently. The immunoblot of the covalently bound fraction also indicated that cells covalently anchored Ccw12-β-A much more efficiently than Ccw12-β-B. However, both Ccw12-β fusion proteins were anchored less efficiently than Pir2-Δ7 and Pir2-Δ1. Thus, although both Ccw12-β-lactamase fusion proteins were secreted and C-terminally anchored to the cell surface, Ccw12-β-A performed better.

Next, we compared the enzymatic activities of Ccw12-β-A and Ccw12-β-B with those of Pir2-Δ7 and Pir2-Δ1 (Fig. 2D). There was no significant difference in activity between Ccw12-β-A and Ccw12-β-B (*p*-value = 0.924, Welch two sample *t*-test), indicating that the 3β-strand motif does not impact the Ccw12-based surface display. However, the Ccw12-β-lactamase reporters performed significantly better than Pir-tagged Pir2-Δ7 (1.28-fold increase, *p*-value = 0.0168, Welch two sample *t*-test), although immunoblotting indicated that the latter was more efficiently anchored to the cell surface. This discrepancy could stem from a large amount of Ccw12-β-A and Ccw12-β-B being present in the non-covalently bound fraction of the cell wall or from potential steric hindrances affecting β-lactamase activity when the enzyme is anchored at the N-terminus. Nonetheless, the most efficient Pir2-based reporter, Pir2-Δ1, still outperformed the Ccw12-β-lactamase reporters (1.46-fold increase, *p*-value = 10^− 6^, Welch two sample *t*-test). Thus, we successfully constructed a functional Ccw12-based system for C-terminal anchoring on the cell’s outer surface, comparable in efficiency to the previously developed Pir2-based surface display for N-terminal anchoring.

The preceding results enabled us to identify the Ccw12-tag, i.e., the minimal portion of the Ccw12 protein necessary to efficiently anchor a protein’s C-terminus to the outer cell surface. Based on the Ccw12-β-A design, this bipartite 50-amino acid tag comprises the Ccw12 signal sequence (amino acids 1–20) and the Ccw12 tether (amino acids 104–133), which need to be appended to the N- and C-terminus of the target protein, respectively. While the tag weighs 4.6 kDa, after cleavage of the N-terminal signal sequence and processing by GPI transamidase, the molar mass of the resulting protein increases by only 1.1 kDa.

### Eukaryotic glucosyl glycerol production through C-terminal anchoring of *L. mesenteroides* SucP

**Fig. 3:**
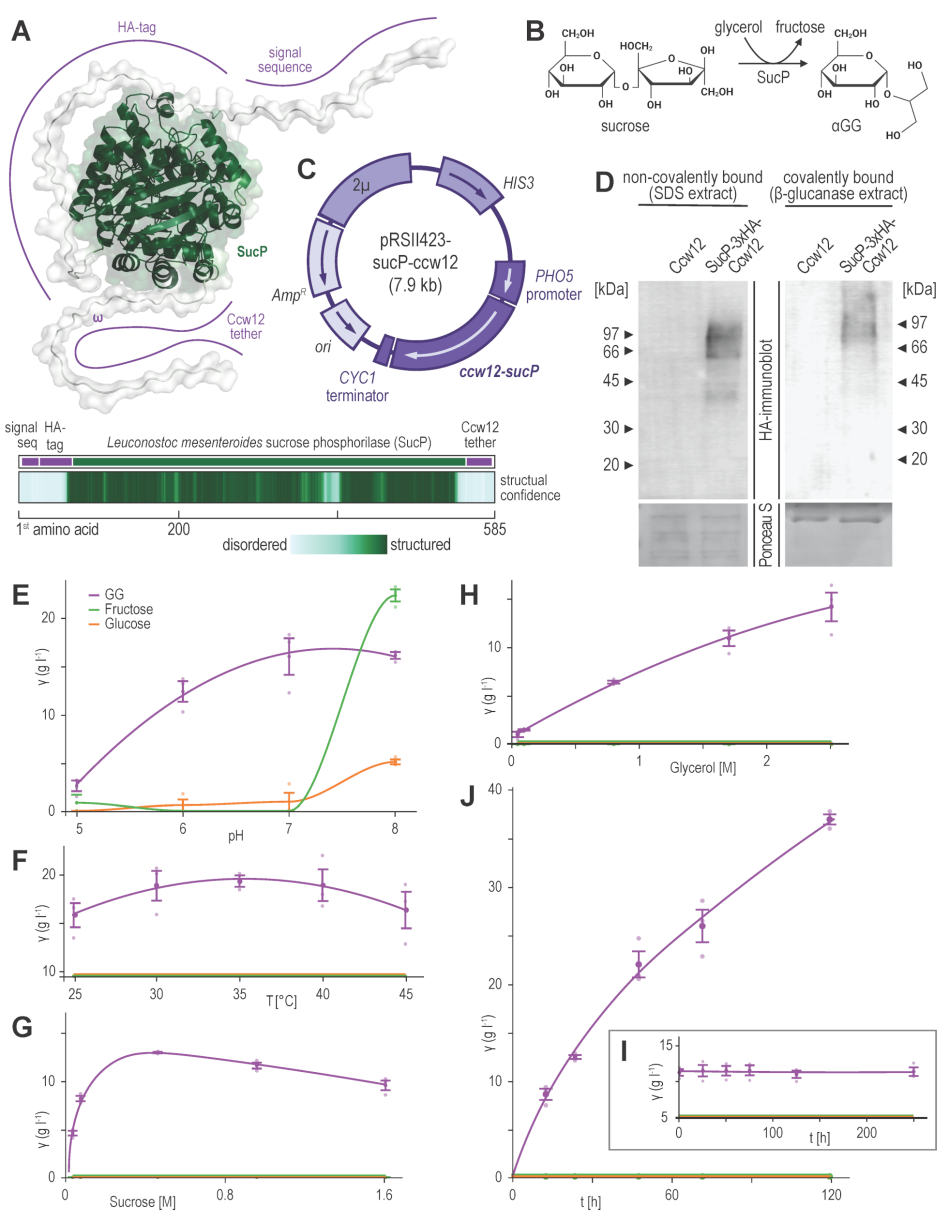
Sucrose phosphorylase (SucP) fused with a minimal Ccw12-tag. (**A**) The predicted 3D structure of the fused protein, colour-coded by the plDDT score, highlighting structural features. Below the structure, the tracks correlate these features with their positions in the primary sequence and corresponding plDDT scores. (**B**) The reaction through which SucP produces αGG. (**C**) Map of a yeast shuttle vector encoding the C-terminally anchored SucP. *ccw12-sucP* – gene encoding SucP fused with the minimal Ccw12-tag. The remaining labels are defined in Fig. 2. (**D**) Anti-HA immunoblotting of C-terminally anchored SucP, with the lower Ponceau S-stained membrane serving as a loading control. (**E**) The concentrations of αGG and monosaccharide by-products after a 20-hour incubation at different pH values, (**F**) at different incubation temperatures, (**G**) with varying initial concentrations of sucrose and (**H**) glycerol, (**I**) after up to 10 days of storing induced cells at 4 °C, and (**J**) during a continuous 5-day reaction. On graphs F-J, lines denoting glucose and fructose concentrations largely overlap, due to the complete absence of both monosaccharides

We then used the minimal Ccw12-tag to C-terminally anchor SucP from *Leuconostoc mesenteroides* DSM 20,193 (Fig. 3A) and thus extracellularly produce αGG (Fig. 3B). We fused the Ccw12 signal sequence, a triple HA-tag, and a His-tag to the *L. mesenteroides* open reading frame (ORF) encoding SucP (amino acids 2-490), and followed it by Ccw12 tether (Ccw12 amino acids 104–133). The resulting ORF, encoding 65.3 kDa C-terminally tethered SucP, was placed on 2µ *HIS3* yeast shuttle vector, under the inducible *PHO5* promoter and strong *CYC1* terminator (Fig. 3C). The plasmid was transformed into *S. cerevisiae* strain SEY 6210, which lacked functional invertase Suc2 and thus could not unproductively hydrolyse the SucP substrate sucrose to glucose and fructose. Growth rate comparisons between the parental SEY 6210 strain and its SucP-encoding derivative revealed no significant differences (data not shown).

To confirm that the Ccw12-tagged SucP was indeed secreted and C-terminally anchored on the cell surface, we performed an anti-HA immunoblot of the non-covalently and covalently bound cell wall protein fractions (Fig. 3D). The immunoblot showed that Ccw12-SucP successfully traversed the *S. cerevisiae* secretion system and became C-terminally anchored on the outer cell surface. This observation stemmed from the diffuse immunoblotting signals above 66 kDa in both SDS and β-glucanase protein fractions, which likely indicate extensive N-glycosylation, given that SucP carries four putative N-X-T/S glycosylation sites (N343, N427, N469, and N486). Thus, the Ccw12-tagged SucP was successfully secreted and C-terminally anchored to the cell wall.

Next, we verified the functionality of the surface-displayed SucP. During an initial 20-hour incubation in a solution containing 284 g l^− 1^ (0.8 M) of sucrose and 177 g l^− 1^ (1.9 M) of glycerol, induced yeast cells (20 OD_600_/ml) produced up to 9.5 g l^− 1^ (37 mM) of αGG, while consuming 6.7 g l^− 1^ (73 mM) of glycerol and 23.1 g l^− 1^ (67 mM) of sucrose. Throughout the reaction, the cells metabolised glucose and fructose generated by spontaneous and SucP-catalysed sucrose hydrolysis, as well as the fructose released during the glucosylation reaction, which could simplify downstream processing. Thus, surface-displayed SucP was catalytically active and capable of producing significant quantities of the natural osmolyte αGG.

We also examined the kinetic parameters of the surface-displayed SucP. The tethered enzyme had a pH optimum of 7 (Fig. 3E), which aligned closely with that of the free enzyme [7, 43]. At higher pH values, the cells failed to metabolise all the available glucose and fructose. Specifically, during 20 h at pH 7, cells produced 17.9 g (70.4 mM) of αGG by metabolising 41.3 g (120.65 mM) of sucrose and 9.2 g (99.9 mM) glycerol, while 223.0 g (0.65 M) of sucrose and 170.9 g (1.86 M) of glycerol remained in the reaction mixture. No leftover glucose or fructose was detected, suggesting cells metabolised all monosaccharides released through spontaneous or enzymatic lysis of sucrose. Conversely, during 20 h at pH 8, cells produced 16.1 g (63.3 mM) of αGG by metabolising 33.4 g (97.6 mM) of sucrose and 11.7 g (127.0 mM) glycerol, while 240.7 g (0.7 M) of sucrose and 176.5 g (1.92 M) of glycerol remained in the reaction mixture, as well as 5.1 g (28.3 mM) of glucose and 22.4 g (12.4 mM) of fructose. Such results align with yeast’s acidophilic nature and previously noted effects of high pH on its metabolism [44, 45].

The enzyme exhibited a broad temperature optimum, ranging from 30 to 40 °C (Fig. 3F), K_M_ values for sucrose and glycerol of 0.07 M and 0.55 M, respectively (Fig. 3G and H). However, the reaction temperature significantly affected cell metabolism and, thus, yield. While at 25 °C, 35 °C, and 45 °C cells produced 17.0 g (66.9 mM), 20.4 g (80.2 mM), and 17.5 g (68.8 mM) of αGG, they metabolised 29.4 g (85.9 mM), 50.0 g (146.1 mM), and 34.9 g (102.0 mM) of sucrose and 6.2 g (67.3 mM), 10.9 g (118.4 mM), and 4.5 g (48.8 mM) of glycerol, respectively. As such, carrying out the reaction at a lower bound of the optimal temperature range is likely preferable.

Finally, the catalytic efficiency of SucP displayed on the cell surface did not decrease when the cells were pelleted and stored at 4 °C for up to 10 days before starting the reaction (Fig. 3I). After determining enzymatic parameters, we followed the reaction for up to 5 days in 0.7 M sucrose and 2.1 M glycerol. During this time, the concentration of αGG rose near-linearly, with the 20 OD_600_ cells ml^− 1^ metabolising 126.21 g l^− 1^ (369 mM) of sucrose and 16.51 g l^− 1^ (180 mM) of glycerol to produce 37.3 g l^− 1^ (146 mM) of αGG (Fig. 3J).

## Discussion

In this work, we transformed yeast *S. cerevisiae* into the first eukaryotic microbial cell factory capable of a one-step αGG production from the readily available substrates sucrose and glycerol. This system employed *L. mesenteroides* SucP, which can use glycerol as a glucosyl acceptor in a low-water, glycerol-rich, phosphate-free environment - conditions that are incompatible with intracellular metabolism. We addressed this incompatibility by employing surface display methodology and further stabilized enzymatically active SucP on the outer cell surface by covalently binding it to the cell wall’s β-1,6-glucan using the newly developed minimal Ccw12-tag. Moreover, we leveraged highly-developed genetic tools for *S. cerevisiae* to tailor the SucP genetic construct and the yeast chassis to the reaction conditions, expressing the SucP isoform in an invertase-negative strain, from the *PHO5* promoter, i.e., a promoter strongly induced under phosphate-free conditions.

We based our system for the C-terminal surface display on the structure of Ccw12, a highly abundant cell wall glycoprotein. We previously developed a corresponding Pir-tag, an N-terminal peptide that directs proteins to the cell wall and covalently anchors them to the outer cell surface near their N-terminus [15]. However, as not all proteins remain functional after N-terminal immobilization, we here presented the corresponding tag for C-terminal immobilization. Through structural analysis of Ccw12, we derived a minimal, bipartite Ccw12-tag comprising 50 amino acid residues. After processing in the secretory pathway, this tag adds only 1.1 kDa to the secreted protein while enabling covalent C-terminal anchoring of targeted protein to β-1,6-glucan.

While developing a minimal Ccw12-tag, we uncovered that Ccw12 encodes a well-structured 3β-strand motif, also present in several other cell wall proteins. The motif likely supports the formation of Ccw12 homodimers and -trimers, as well as the docking of Ccw12 to other 3β-strand cell wall proteins, such as flocculins. This hypothesis explains the uneven distribution of Ccw12 on the cell wall [35], as, according to it, Ccw12 would gather around other 3β-strands proteins. Once formed, such nucleation points would encourage further formation of Ccw12 hubs, i.e., the formation of transient 3β-strand heterodimers and C72-C72 disulphide bond-supported Ccw12 homodimers and -trimers. As Ccw12 is highly N-glycosylated, such Ccw12 hubs would enrich their surroundings with N-glycans, presumably strengthening them. Thus, we propose that Ccw12 functions as a glycan-rich adapter protein that nucleates around other 3β-strand proteins, forming Ccw12 hubs that reinforce newly synthesized, malleable regions of the cell wall.

The here described minimal bipartite Ccw12-tag overcomes the limitations of previous Ccw12-based surface displays. These earlier versions of the Ccw12-based surface display [46, 47] did not consider the structural aspects of Ccw12, focusing instead on convenient restriction sites within the *CCW12* open reading frame. This approach constrained protein design and prevented analysis of how specific Ccw12 regions affect surface display. By adopting a structure-oriented approach, we identified functional sections of Ccw12 and opted to retain or exclude them. Thus, we designed fusion proteins lacking the N-glycosylated, Ccw12-characteristic repeats R1 and R2. This reduction in glycosylation ensured the proteins’ visibility on a resolving polyacrylamide gel, providing a more effective method for studying Ccw12-based surface display. Moreover, through Ccw12-β-A and Ccw12-β-B constructs, we investigated how the 3β-strand motif influenced Ccw12-based surface display. While the nitrocefin assay showed the motif had no effect on β-lactamase activity, immunoblotting revealed it promoted heterogeneous glycosylation, possibly by interacting with the glycosylation machinery of the secretory pathway.

The 3β-strand motif also influenced the covalent binding of fusion proteins to the cell wall. Fewer fusion proteins with the 3β-strand motif were covalently bound to the cell surface, possibly due to the motif’s tendency to form protein-protein complexes. Once secreted to the cell wall, the fusion protein could either integrate into the non-covalently bound mannoprotein layer or be processed by GPI transamidase, which would covalently tether it to β-1,6-glucan via a GPI anchor. Thus, the complex-forming nature of the 3β-strand motif may be directing Ccw12-β-B more efficiently toward the non-covalently bound mannoproteins, before GPI transamidase can act, thus preventing their covalent tethering.

The Ccw12- and Pir-tags serve distinct purposes, with the former designed for C-terminal anchoring and the latter for N-terminal anchoring. Thus, for successful protein immobilization, the Ccw12-tag needs to be placed at the protein’s C-terminus, as any sequence following the glycine residue in the GPI signal will be cleaved off and fail to immobilize on the cell wall. However, it is still possible to compare Ccw12- and Pir-tags, using β-lactamase as a reporter enzyme that can tolerate both N- and C-terminal anchoring. Thus, the nitrocefin assay showed the Ccw12-tag more effectively displayed β-lactamase on the cell surface, possibly because Pir proteins are partially secreted via an alternative secretion pathway, as suggested by previous studies [48–50]. The assay also showed the Pir-construct with an extended unstructured region outperformed the Ccw12-tag. This finding implies that adding a similar unstructured region into the Ccw12-tag might boost its efficacy.

We practically applied the Ccw12-tag by using it to C-terminally anchor sucrose phosphorylase (SucP) from *Leuconostoc mesenteroides*. This enzyme acts upon sucrose and catalyses its phosphorolysis, i.e., glucosyl transfer to and from phosphate, as well as sucrose’s hydrolysis and transglycosylation in the absence of phosphate. As such, the immobilized enzyme has previously been used for efficient one-step αGG production from a highly concentrated phosphate-free solution of sucrose and glycerol [51]. While the bioprocess based on densely-packed chemically immobilized SucP is highly productive [9], it is also more costly and time-consuming compared to surface display.

Once fused with the Ccw12-tag, SucP reached the cell surface and remained active, producing αGG despite likely being N-glycosylated at up to four potential N-X-T/S sites, as evidenced by the smear on the immunoblot. As glycosylation is known to stabilize the enzyme structure or shield it from environmental stress [52, 53], it might have helped preserve the enzyme’s activity during reactions that spanned several days and during extended storage at 4 °C.

In the presented bioprocess, yeast cells did more than serve as an immobilization platform for SucP: they remained metabolically active, adding significant value to the process. The cells metabolized SucP’s transglycosylation and hydrolysis by-products: fructose, which is difficult to remove during αGG purification due to its chromatographic characteristics [54], and glucose, a by-product of non-productive reactions where the glucosyl moiety is transferred to water rather than glycerol [7]. Throughout the bioprocess, the cells remained viable and continued their metabolic activity for up to five days, as would be expected of non-dividing yeast cells fed ad libitum [55]. Furthermore, the sustained SucP activity suggests the cells continually renewed the enzyme on their surface and implies the importance of using appropriate promoters strongly induced during long-term bioprocesses, e.g., *PHO5* promoter compatible with the environment required by the SucP reaction. Thus, throughout the entire reaction, the cells remained metabolically active, fuelled by the carbon sources abundant in the reaction mixture: sucrose and glycerol. Indeed, throughout the 5-day incubation, yeast used only 40% of spent sucrose for synthetising αGG. However, although the yeast consumed the SucP substrates sucrose and glycerol, lowering overall productivity, the low cost of these substrates means their consumption does not substantially affect the bioprocess viability. Of course, in industrial applications, additional challenge would be to remove unutilised sucrose and glycerol. Ultimately, the yeast surface display of SucP effectively utilized the cells’ metabolic activity to simplify downstream processing and extend viable reaction time.

Yeast cells also shaped the kinetic parameters of the surface-displayed SucP and the reaction mixture’s final composition. The surface-displayed SucP had similar pH and temperature dependencies as the free enzyme [7]. However, when the pH was raised to 8, a value less conducive to yeast metabolic activity [44], the cells metabolised glucose and fructose less effectively. Moreover, although SucP is not inhibited by its substrates, the αGG production was slowed at high sucrose concentrations, likely due to osmotic stress that affected cell homeostasis and thus reduced the effective surface display. This observation further confirms the cells remain responsive to their surroundings even in a harsh, high sucrose-glycerol environment. In contrast, the cells coped well with high concentrations of glycerol, with the data suggesting that increasing glycerol beyond 2.5 M would boost αGG production further. This result aligns with SucP’s two-step catalytic mechanism, which favours glycerol and water as a glucosyl acceptor in the absence of phosphate [7] and thus facilitates higher αGG yield in glycerol-rich water-poor environments. Thus, the characteristics of the implemented surface display are determined both by the enzyme displayed and the microbial chassis used to implement the system.

## Conclusion

In this study, we introduced the first eukaryotic microbial cell factory capable of one-step αGG production. As the key enzyme SucP catalysed the required reaction under conditions incompatible with standard intracellular metabolism, we relocated it to the outer cell surface and further stabilized it through a novel minimal Ccw12-tag that efficiently C-terminally anchored SucP to the cell surface. The here presented strategy could also advance other bioprocesses reliant on enzymatic reactions incompatible with typical intracellular conditions.

## Electronic supplementary material

Below is the link to the electronic supplementary material.

Supplementary Material 1: Details of the plasmid and strain construction

Supplementary Figure 1: Alphafold 3 modelling of the Aga1-Aga2-Ccw12 complex. The structure is colour-coded to reflect local confidence scores per amino acid residue (plDDT), with light green, blue, and purple indicating low confidence and dark colours indicating high confidence in Ccw12, Aga1, and Aga2 structures, respectively. The red triangle marks the C72-C111 Ccw12-Aga1 interstrand disulphide bond and the purple triangles Aga1-Aga2 disulphide bonds

## Data Availability

No datasets were generated or analysed during the current study.
