# Supplementary figures and images for: Engineering Saccharomyces cerevisiae for the production of natural osmolyte glucosyl glycerol from sucrose and glycerol through Ccw12-based surface display of sucrose phosphorylase
Source: J Biol Eng. 2024 Nov 22;18:69. doi: 10.1186/s13036-024-00468-7 (PMC11583750; doi:10.1186/s13036-024-00468-7)

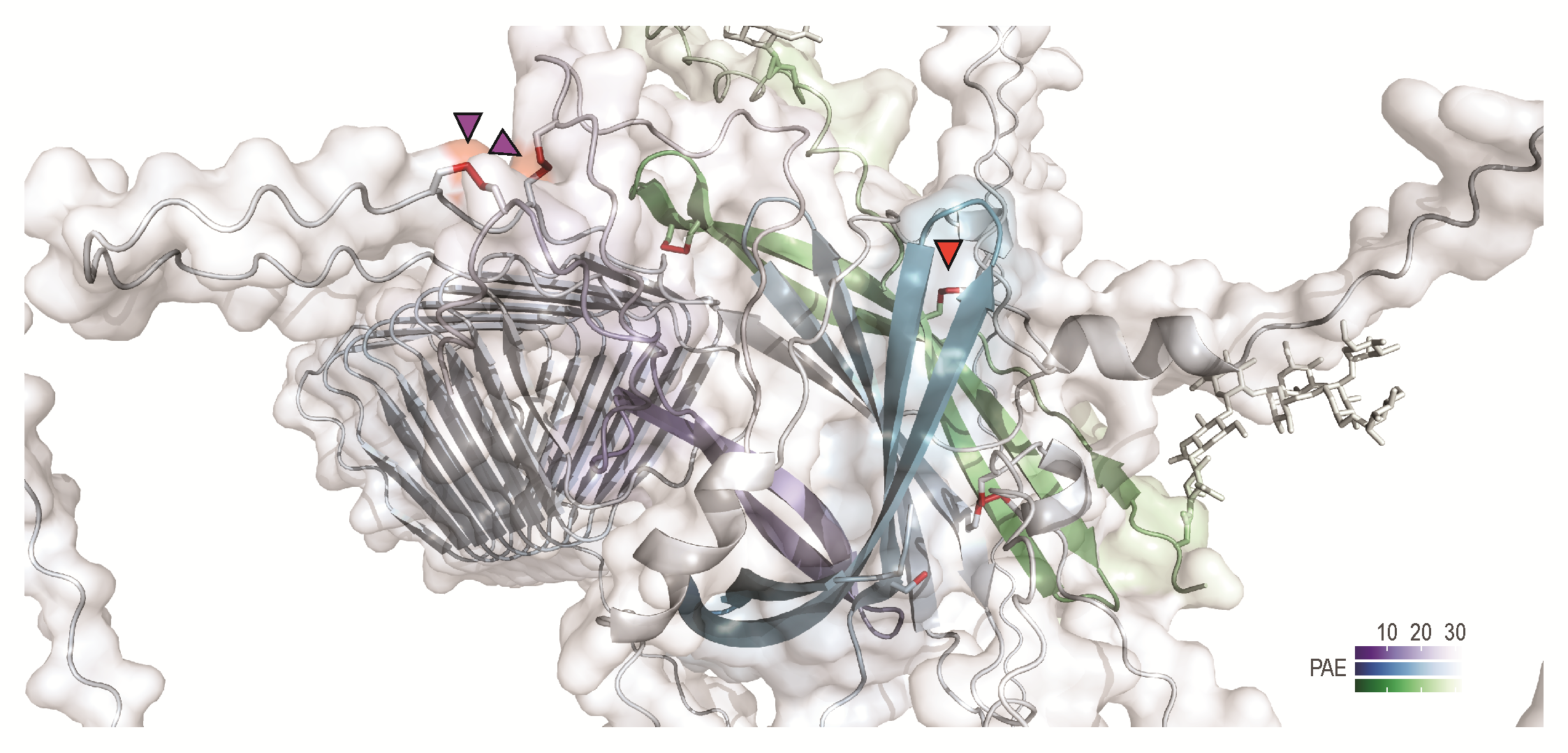

Supplement: Supplementary file 2 — Supplementary Figure 1: Alphafold 3 modelling of the Aga1-Aga2-Ccw12 complex. The structure is colour-coded to reflect local confidence scores per amino acid residue (plDDT), with light green, blue, and purple indicating low confidence and dark colours indicating high confidence in Ccw12, Aga1, and Aga2 structures, respectively. The red triangle marks the C72-C111 Ccw12-Aga1 interstrand disulphide bond and the purple triangles Aga1-Aga2 disulphide bonds [file 13036_2024_468_MOESM2_ESM.png]
